# Determination of Length of Interdigitation Zone by Optical Coherence Tomography and Retinal Sensitivity by Microperimetry and Their Relationship to Progression of Retinitis Pigmentosa

**DOI:** 10.1155/2019/1217270

**Published:** 2019-06-20

**Authors:** Akihiro Chiba, Gen Miura, Takayuki Baba, Shuichi Yamamoto

**Affiliations:** Department of Ophthalmology and Visual Science, Chiba University Graduate School of Medicine, Chiba, Japan

## Abstract

**Purpose:**

To investigate the annual progression of retinitis pigmentosa (RP) by changes in retinal sensitivity and length of photoreceptor microstructures.

**Method:**

The medical records of patients with typical RP followed at Chiba University Hospital were reviewed. The retinal sensitivity was measured by Micro Perimeter-1, and the lengths of the intact external limiting membrane (ELM), ellipsoid zone (EZ), and interdigitation zone (IZ) were measured by spectral-domain optical coherence tomography. The baseline values and annual progression rates were determined. The significance of the correlations among these factors was determined by generalized estimating equation regression analysis.

**Results:**

Forty-six eyes of 24 patients who were examined over a mean follow-up period of 3 years were studied. The annual changes in the retinal sensitivity (*p* = 0.0035) and the lengths of the EZ (*p* = 0.037) and IZ (*p* = 0.0033) were significantly correlated with their baseline values. The annual change in the retinal sensitivity was significantly correlated with the length of the EZ at the baseline (*p* = 0.020).

**Conclusions:**

The significant correlation between the annual progression of the retinal sensitivity and the baseline retinal sensitivity and lengths of the EZ and IZ in patients with RP indicate that the retinal sensitivity, the EZ, and the IZ can be useful parameters to predict the annual progression of RP.

## 1. Introduction

Retinitis pigmentosa (RP) is an inherited retinal disorder that is associated with progressive degeneration of the photoreceptors and subsequent irreversible reduction of vision. RP affects approximately 1 in 4000 individuals worldwide, and RP patients typically present with night blindness followed by a constriction of the visual fields and severe visual impairment and blindness [1, 2]. Genetic counseling and instructions on optimizing the residual vision remain the main methods of managing RP as there are currently few treatments that can stop or reverse the progression of RP [3, 4]. However, there are a number of therapeutic trials for RP such as gene- [4], drug- [5], and cell-based therapy [6]. Therefore, determining the natural course of the degeneration of the retinal structure and visual function in eyes with RP is important for determining the effectiveness of any type of therapy.

The results of earlier studies have shown that there are significant associations between the visual function and the integrity of the outer retinal bands, such as the length of the external limiting membrane (ELM) [7, 8], the ellipsoid zone (EZ) [7–12], and the interdigitation zone (IZ) [8]. In addition, several studies have shown that the EZ determined by spectral-domain optical coherence tomography (SD-OCT) was a sensitive and reliable marker for assessing the progression of RP [13–19]. In addition, Hagiwara et al. [8] reported that the lengths of the ELM, EZ, and IZ were significantly correlated with the visual acuity and retinal sensitivity. They reported that their lengths were correlated with each other, and the IZ was the first microstructure to become disrupted followed by the EZ and finally the ELM in eyes with RP. In spite of these early findings, the progression of ELM and IZ and the relationship between the progression of retinal sensitivity and the outer retinal bands has not been definitively determined.

Thus, the purpose of this study was to evaluate the annual progression of the retinal sensitivity and the length of the ELM, EZ, and IZ and to determine the relationships between the baseline status and the annual progression in eyes with RP.

## 2. Materials and Methods

This was a review of the medical records of patients with typical RP whose retinal sensitivity and retinal tomographic images had been recorded for at least 2 years at the Chiba University Hospital. The procedures used in this study were approved by the Institutional Review Board of Chiba University (0314), and they conformed to the tenets of the Declaration of Helsinki. An informed consent had been obtained from the patients at the time of their examinations to use their medical information for research studies. Assurance was provided that the anonymity of each patient would be preserved. The diagnosis of RP was based on the clinical history, funduscopic appearance, visual field testing, fluorescein angiography, and full-field electroretinograms (ERGs) recorded according to International Society for Clinical Electrophysiology of Vision (ISCEV) standardized conditions. Typical RP was defined as RPs excluding atypical RP, such as sector RP and unilateral RP. Patients were also excluded if they had high myopia, an epiretinal membrane, macular edema, poor fixation, cataract in the center of the lens that affected the retinal sensitivity, and the presence or absence of the entire ELM, EZ, and IZ determined by OCT at the baseline. We determined the inheritance pattern of the participants from the family history.

The best-corrected visual acuity (BCVA) was measured monocularly using a Japanese standard Landolt ring chart (System Charts SC-2000 Nidek Instruments, Gamagori, Japan) at a distance of 5 meters. The decimal visual acuities were converted to the logarithm of the minimum angle of resolution (logMAR) units for the statistical analyses.

**Figure 1:**
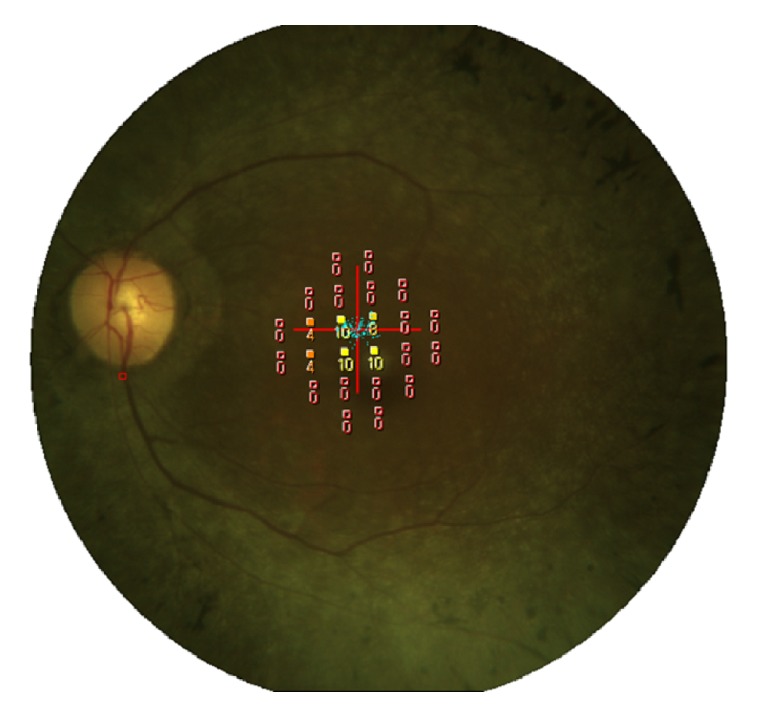
Micro Perimeter-1 image. A total of 24 stimulus locations covering the central 10° field were tested.

The retinal sensitivity was determined by Micro Perimeter-1 (MP-1, Nidek Co. Ltd., Aichi, Japan) at 24 locations in the central 10 degrees of an extracted Humphrey Field Analyzer 10-2 pattern, and the mean retinal sensitivity of the 24 locations was calculated and used for the statistical analyses (Figure 1).

**Figure 2:**
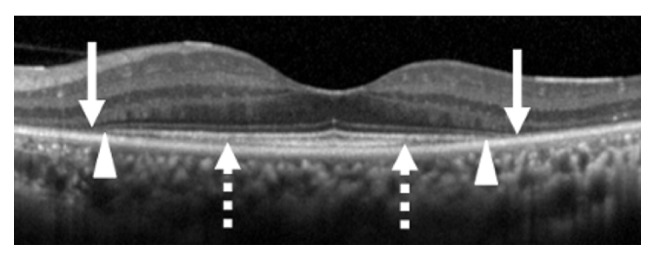
Optical coherence tomographic image centered on the fovea of a patient with RP. Arrows indicate the ends of the external limiting membrane line, arrowheads indicate the ends of the ellipsoid zone line, and dashed arrows indicate the end of the interdigitation zone.

The integrities of the outer retinal bands were determined by the SD-OCT images which were obtained with the Spectralis OCT (Heidelberg Engineering, Heidelberg, Germany) from 9 mm horizontal and vertical scans of 100 averaged images through the fovea (Figure 2). Only OCT scans of good quality (higher than 25 dB) were used for the measurements.

The average of the horizontal and vertical lengths of the ELM, EZ, and IZ was used for the statistical analyses. The ELM, EZ, and IZ were measured independently by two of the authors (AC and GM) in a masked way. In the event of disagreement, the two graders examined the images together, and the final length of the lines was decided.

For the statistical analyses, the annual progression in the retinal sensitivity, ELM, EZ, and IZ were defined as the values calculated by dividing the difference between the value at the baseline and the value at the final examination by the number of years between the examinations. The annual progression rate was defined as the annual progression value/baseline value. Statistical analysis was performed using the R statistical software package, version 3.4.1. Paired* t*-tests were used to determine the significance of the changes in the values during the observation period. The associations between the baseline values and the annual decrease were determined using the generalized estimating equations (GEE) regression analysis because of the intereye correlations. The marginal R^2^ (mR^2^) was calculated to measure the proportion of variance. The level of significance was set as* p* <0.05.

## 3. Results

Forty-six eyes of 24 patients (7 men and 17 women) were studied. The mean age ± standard deviation (SD) of the patients at the baseline was 56.9 ± 11.7 years. The mean BCVA was 0.0420 ± 0.120 logMAR units at the baseline and was 0.0867 ± 0.165 logMAR units at the final examination. The mean interval between the baseline and the final examination was 36.9 ± 4.7 months. The inheritance pattern was autosomal dominant, 2 eyes of one patient; autosomal recessive, 4 eyes of 2 patients; and sporadic, 40 eyes of 21 patients. The clinical findings of the 46 eyes are shown in Table 1. There was a significant decrease in the retinal sensitivity (*p* <0.0001, paired* t*-test), the ELM length (*p* <0.0001), the EZ length (*p* <0.0001), and the IZ length (*p* <0.0001) from the baseline to the final examination.

The annual progression and progression rate are shown in Table 2. The mean annual progression in the retinal sensitivity was 0.880 ± 0.756 dB/year (7.4 ± 7.6%/year), the ELM was 90.9 ± 110 *μ*m/year (2.0 ± 2.2 %/year), the EZ was 143 ± 136 *μ*m/year (3.8 ± 3.3 %/year), and the IZ was 172 ± 198 *μ*m/year (12 ± 16 %/year).

The correlations between the annual progression rates and baseline values of each parameter are shown in Table 3. The annual progression rate in the retinal sensitivity was significantly correlated with the retinal sensitivity at the baseline (*p* = 0.0035, mR^2^ = 0.020, GEE). The annual decrease in the length of the ELM was not significantly correlated with the length at the baseline (*p* = 0.82), but the annual decrease in the length of the EZ (*p* = 0.037, mR^2^ = 0.16) and the IZ (*p* = 0.0033, mR^2^ = 0.19) was significantly correlated with their baseline values.

The correlations between the annual progression rate of the retinal sensitivity and the baseline values of the outer retinal bands are shown in Table 4. The lengths of the ELM and IZ at the baseline were not significantly correlated with the annual progression rate in the retinal sensitivities (*p* = 0.47,* p* = 0.86, GEE, respectively), but the length of the EZ at the baseline was significantly correlated with the annual progression rate in the retinal sensitivity (*p* = 0.020, mR^2^ = 0.11).

## 4. Discussion

The relationships between the annual progression of retinal sensitivity and the lengths of the ELM, EZ and IZ of RP patients were investigated. Our findings showed that a poorer retinal sensitivity and shorter lengths of the EZ and IZ at the baseline were significantly correlated with a slower decrease in the retinal sensitivity and slower decrease in the lengths of the EZ and IZ. In addition, the annual decrease of retinal sensitivity was significantly correlated with the length of the EZ at the baseline.

The mean decrease of the EZ length was 143 *μ*m/year (3.8%/year) which was comparable with previous studies that ranged from 3.4 to 9.6%/year or from 76.4 to 140 *μ*m/year [14, 17–19]. Cabral et al. [17] reported that the progression rates were slower for patients with EZs that were ≤3000 *μ*m at the baseline. In addition, Colombo et al. [18] reported that the progression rate of the EZ decreased when the margins of the atrophic retina approach the foveal region. In our study, a shorter baseline length was associated with the slower progression. Thus, our results are in good agreement with the earlier findings.

We found that the lengths of not only the EZ but also the IZ and retinal sensitivity were significantly correlated with the degree of their annual progression rate. To the best of our knowledge, there have not been any reports on the annual progression rates of the IZ in RP patients. Hagiwara et al. [8] reported that the length of the IZ was correlated with the BCVA and retinal sensitivity. In addition, previous studies reported that the IZ contributed more to the degree of reflectance of the photoreceptor mosaic than the EZ in the adaptive optics images in retinal diseases [20–24]. The absence of the IZ in the OCT images could be the earliest sign of a loss of the normal cone mosaics in the adaptive optics images [25]. Thus, there is a possibility that measurements of the IZ will be helpful in assessing the integrity of the photoreceptors in patients with RP as with other retinal diseases.

Kominami et al. [26] reported on the importance of the integrity of the IZ in eyes with rhegmatogenous retinal detachment (RRD). Their study showed that a restoration of the EZ accompanied by that of the IZ was essential for the recovery of the focal macular ERGs after fovea-off RRD. Thus, a restoration of the EZ alone is not enough to improve the focal macular ERGs. In retinal regenerative therapy, a retinal detachment is created for creating space between photoreceptor outer segments and the RPE to deliver a transplant [27]. The IZ will be an important biomarker in future therapies of RP.

Several studies have reported there is a significant association between the retinal sensitivity measured by microperimetry and the retinal structure in patients with RP [7, 8, 10, 12]. MP-1 has been used to determine the retinal sensitivities in patients with macular diseases, and the changes in the sensitivities have been shown to be correlated with the changes in the ophthalmoscopic appearance of the retina [21, 22]. MP-1 is good method to determine the retinal sensitivities of subjects with unsteady fixation or those who have developed a nonfoveal preferred retinal locus. In addition, MP-1 has the advantage of having greater sensitivity in detecting the changes in RP patients than the Humphrey Field Analyzer [28]. Our results revealed that the progression of retinal sensitivity was correlated with the baseline value of the retinal sensitivity and the length of EZ. Thus, microperimetry may be helpful in assessing retinal sensitivities for clinical studies in eyes with RP.

Our study has some limitations. First, our study examined RP patients only at a limited stage of RP. Neither the early stage patients in whom the degeneration has not reached the posterior pole nor the end stage patients in whom the degeneration has reached fovea at the baseline were examined. In those patients, the progress rate may differ from this study. Second, en face OCT images of the retinal and choroidal layers were not examined. Hariri et al. [16] reported that measuring the EZ area in en face OCT images rather than the width on OCT B-scan may be a better method to determine the structure-function correlations because the preserved EZ area may be compared more easily with the preserved island of vision from perimetric assessments. Newer OCT techniques such as enhanced depth imaging OCT and swept source OCT will be needed to measure wider areas of the retinal outer layer. Actually, we recently showed that the luminal area and the ratio of luminal/total choroidal area in the inner choroid were significantly correlated with the visual function in RP patients and that the choroidal structures were altered in association with the progression of RP [29].

Another limitation is the lack of the genetic testing of the participants in this study. The genetic heterogeneity of RP makes the interpretation of the natural progression studies difficult to interpret. In general, the rate of decline in visual function has been attributed to many factors including the mutated gene and type of mutations as well as other genetic and environmental factors [1, 2]. In fact, Cai et al. [14] reported that the progression rate of the EZ length is faster in x-linked RP than autosomal dominant RP. Due to the genetic heterogeneity of RP, further studies on the natural progression in large cohorts and multicenter studies are needed.

In conclusion, the annual progression of the retinal sensitivity and the length of the EZ and IZ were significantly correlated with their baseline values in patients with RP. In addition, the annual progression of the retinal sensitivity was significantly correlated with the length of the EZ at baseline. The retinal sensitivity, the EZ, and the IZ could be candidates for predicting the progression of RP.

## Figures and Tables

**Table 1:** Values of the parameters at the baseline and final examination.

	Baseline	Final	*p* value
Retinal sensitivity, dB	12.7 ± 4.65	10.1 ± 4.56	< 0.0001^*∗*^
ELM, *μ*m	4966 ± 1977	4682 ± 1879	< 0.0001^*∗*^
EZ, *μ*m	4171 ± 1924	3725 ± 1781	< 0.0001^*∗*^
IZ, *μ*m	1576 ± 1111	1033 ± 976	< 0.0001^*∗*^

Data are the means ± SD. *∗*Paired *t*-test. ELM, external limiting membrane; EZ, ellipsoid zone; IZ, interdigitation zone.

**Table 2:** Annual progression of the parameters.

	Annual progression (Progress rate)
Retinal sensitivity (dB/year, %/year)	0.880 ± 0.756 (7.4 ± 7.6)
ELM (*μ*m/year, %/year)	90.9 ± 110 (2.0 ± 2.2)
EZ (*μ*m/year, %/year)	143 ± 136 (3.8 ± 3.3)
IZ (*μ*m/year, %/year)	172 ± 198 (12 ± 16)

Data are the means ± SD. ELM, external limiting membrane; EZ, ellipsoid zone; IZ, interdigitation zone.

**Table 3:** Association between the annual progression and baseline value of each parameter.

	Regression coefficient	Standard error	95% CI	*p* value
Retinal sensitivity	6.20e^−2^	2.12e^−2^	2.04e^−2^ to 1.04e^−1^	0.0035
ELM	1.77e^−2^	1.02e^−2^	-2.28e^−3^ to 3.77e^−2^	0.082
EZ	2.55e^−2^	1.22e^−2^	1.48e^−3^ to 4.95e^−2^	0.037
IZ	9.23e^−2^	3.14e^−2^	3.08e^−2^ to 1.54e^−1^	0.0033

CI, confidence interval; ELM, external limiting membrane; EZ, ellipsoid zone; IZ, interdigitation zone.

**Table 4:** Association between the annual change of retinal sensitivity and the baseline outer retinal bands.

	Regression coefficient	SE	95% CI	*p* value
ELM	5.30e^−5^	7.33e^−5^	-9.07e^−5^ to 1.97e^−4^	0.47
EZ	1.42e^−4^	6.11e^−5^	2.2e^−5^ to 2.62e^−4^	0.020
IZ	-1.38e^−5^	8.12e^−5^	-1.73e^−4^ to 1.45e^−4^	0.86

SE, standard error; CI, confidence interval; ELM, external limiting membrane; EZ, ellipsoid zone; IZ, interdigitation zone.

## Data Availability

The data used to support the findings of this study are available from the corresponding author upon request.
